# Association of Brain Microstructure and Functional Connectivity With Cognitive Outcomes and Postnatal Growth Among Early School–Aged Children Born With Extremely Low Birth Weight

**DOI:** 10.1001/jamanetworkopen.2023.0198

**Published:** 2023-03-02

**Authors:** Sae Yun Kim, Ee-Kyung Kim, Huijin Song, Jung-Eun Cheon, Bung Nyun Kim, Han-Suk Kim, Seung Han Shin

**Affiliations:** 1 Department of Pediatrics, College of Medicine, The Catholic University of Korea, Seoul, Republic of Korea; 2 Department of Pediatrics, Seoul National University College of Medicine, Seoul, Republic of Korea; 3 Department of Radiology, Seoul National University College of Medicine, Seoul, Republic of Korea; 4 Department of Psychiatry, Seoul National University College of Medicine, Seoul, Republic of Korea

## Abstract

**Importance:**

Postnatal growth may be associated with longitudinal brain development in children born preterm.

**Objective:**

To compare brain microstructure and functional connectivity strength with cognitive outcomes in association with postnatal growth among early school–aged children born preterm with extremely low birth weight.

**Design, Setting, and Participants:**

This single-center cohort study prospectively enrolled 38 children 6 to 8 years of age born preterm with extremely low birth weight: 21 with postnatal growth failure (PGF) and 17 without PGF. Children were enrolled, past records were retrospectively reviewed, and imaging data and cognitive assessments occurred from April 29, 2013, through February 14, 2017. Image processing and statistical analyses were conducted through November 2021.

**Exposure:**

Postnatal growth failure in the early neonatal period.

**Main Outcomes and Measures:**

Diffusion tensor images and resting-state functional magnetic resonance images were analyzed. Cognitive skills were tested using the Wechsler Intelligence Scale; executive function was assessed based on a composite score calculated from the synthetic composite of the Children’s Color Trails Test, STROOP Color and Word Test, and Wisconsin Card Sorting Test; attention function was evaluated using the Advanced Test of Attention (ATA); and the Hollingshead Four Factor Index of Social Status–Child was estimated.

**Results:**

Twenty-one children born preterm with PGF (14 girls [66.7%]), 17 children born preterm without PGF (6 girls [35.3%]), and 44 children born full term (24 girls [54.5%]) were recruited. Attention function was less favorable in children with PGF than those without PGF (mean [SD] ATA score: children with PGF, 63.5 [9.4]; children without PGF, 55.7 [8.0]; *P* = .008). Significantly lower mean (SD) fractional anisotropy in the forceps major of the corpus callosum (0.498 [0.067] vs 0.558 [0.044] vs 0.570 [0.038]) and higher mean (SD) mean diffusivity in the left superior longitudinal fasciculus–parietal bundle (8.312 [0.318] vs 7.902 [0.455] vs 8.083 [0.393]; originally calculated as millimeter squared per second and rescaled 10 000 times as mean diffusivity × 10 000) were seen among children with PGF compared with children without PGF and controls, respectively. Decreased resting-state functional connectivity strength was observed in the children with PGF. The mean diffusivity of the forceps major of the corpus callosum significantly correlated with the attention measures (*r* = 0.225; *P* = .047). Functional connectivity strength between the left superior lateral occipital cortex and both superior parietal lobules correlated with cognitive outcomes of intelligence (right superior parietal lobule, *r* = 0.262; *P* = .02; and left superior parietal lobule, *r* = 0.286; *P* = .01) and executive function (right superior parietal lobule, *r* = 0.367; *P* = .002; and left superior parietal lobule, *r* = 0.324; *P* = .007). The ATA score was positively correlated with functional connectivity strength between the precuneus and anterior division of the cingulate gyrus (*r* = 0.225; *P* = .048); however, it was negatively correlated with functional connectivity strength between the posterior cingulate gyrus and both superior parietal lobules (the right superior parietal lobule [*r* = −0.269; *P* = .02] and the left superior parietal lobule [*r* = −0.338; *P* = .002]).

**Conclusions and Relevance:**

This cohort study suggests that the forceps major of the corpus callosum and the superior parietal lobule were vulnerable regions in preterm infants. Preterm birth and suboptimal postnatal growth could have negative associations with brain maturation, including altered microstructure and functional connectivity. Postnatal growth may be associated with differences in long-term neurodevelopment among children born preterm.

## Key Points

**Question:**

How do brain microstructure and functional connectivity in early school–aged preterm children with postnatal growth failure (PGF) differ compared with those in preterm children without PGF?

**Findings:**

In this cohort study of 38 children born with extremely low birth weight (ELBW) and 44 controls born at full term, children born with ELBW with PGF had worse attention function and lower fractional anisotropy in the forceps major, as well as decreased functional connectivity strength between the superior lateral occipital cortex and the superior parietal lobules.

**Meaning:**

This study suggests that PGF is associated with aberrant structural and functional connectivity and neurodevelopmental impairment in early school–aged children.

## Teaser

This cohort study compares brain microstructure and functional connectivity strength with cognitive outcomes in association with postnatal growth among early school–aged children born preterm with extremely low birth weight.

## Introduction

Although the survival rate of preterm infants has improved over the years, children born very preterm are at a higher risk of adverse neurodevelopmental outcomes^1,2,3^ because the third trimester of pregnancy is a critical period for prolific brain development.^4^ Furthermore, postnatal growth failure (PGF) and associated impaired neurodevelopmental outcomes remain significant in terms of morbidity.^5^ If preterm infants experience growth failure during the critical period, brain development and maturation can be altered, resulting in suboptimal neurodevelopmental outcomes.^6,7,8^

Early investigations used conventional magnetic resonance imaging (MRI) and diffusion tensor imaging to identify alterations in cerebral structural development associated with preterm births.^9,10,11^ In a study from Canada, serial diffusion tensor imaging measures of cortical development in infants born very preterm revealed that the development of normal brain structure was associated with postnatal growth.^12^ Another study showed that high intake of calories, protein, and fat during the first 2 weeks after birth was associated with the maturation of preterm infants’ brains at term-equivalent age.^13^ Researchers from the Netherlands found a positive association of nutrition and weight gain with brain volume.^14^ In contrast, another study among preterm infants did not find a significant association of energy and protein intake with brain volume.^15^ To date, there are no consistent findings on the association of postnatal growth with brain development, especially in early school–aged children, to our knowledge. Resting-state functional MRIs map the temporal correlation of low-frequency fluctuations in blood oxygen level–dependent signal changes across the whole brain.^16^ Several studies have investigated altered functional connectivity across the networks of very preterm infants.^17,18,19^ Decreased functional connectivity between resting-state networks has been reported to remain during early childhood, adolescence, and young adulthood among individuals born very preterm.^20,21,22^ However, research on early school–aged children, as well as the association of postnatal growth with functional connectivity of the brain, has rarely been reported, to our knowledge.

The present study aimed to assess whether early postnatal growth of preterm infants is associated with the white matter microstructure, functional connectivity, and cognitive function. The secondary objective was to examine the correlation between brain microstructure or functional connectivity strength (FCS) and cognitive function. Because previous studies have found an association between PGF and worse neurodevelopmental outcomes,^5,6,7,8^ we hypothesized that early postnatal growth is associated with longitudinal brain development.

## Methods

This was a prospective cohort study using retrospectively acquired perinatal data. The study protocol was approved by the Seoul National University Hospital institutional review board. Parents provided written informed consent, and the participating children received a comprehensive explanation of their participation in the study. This study followed the Strengthening the Reporting of Observational Studies in Epidemiology (STROBE) reporting guideline.

### Participants

Children born with extremely low birth weight (ELBW) were recruited from the Seoul National University Children’s Hospital (SNUCH) preterm follow-up cohort. The ELBW group was divided according to their postnatal growth pattern. Postnatal growth failure was defined as a weight below the third percentile for postmenstrual age based on the Fenton growth chart at discharge from the neonatal intensive care unit. Children whose birth weight was below the third percentile or who had severe brain injury, such as grade 3 or 4 intraventricular hemorrhage or periventricular leukomalacia, were excluded. The control group comprised age-matched, typically developing children born at full term (gestational age [GA] ≥37 weeks and birth weight ≥2500 g), and we additionally recruited children from another cohort study conducted at SNUCH. The study population is detailed in the eMethods and eFigure 1 in Supplement 1.

### Study Design and Procedures

At the age of 6 to 8 years, the participating children underwent MRI and a set of neuropsychological tests conducted by psychologists who were unaware of perinatal findings or postdischarge details. Cognitive skills were tested using the Korean version of the Wechsler Intelligence Scale for Children, Fourth Edition (K-WISC-IV) and the Korean Educational Development Institute–Wechsler Intelligence Scale for Children (KEDI-WISC), and executive function (EF) was assessed based on the EF composite score, which was calculated from the synthetic composite of the Children’s Color Trails Test, the STROOP Color and Word Test, and the Wisconsin Card Sorting Test. Attention function was evaluated using the Advanced Test of Attention (ATA). The Hollingshead Four Factor Index of Social Status estimated the socioeconomic status of children.^23^ Enrollment, image scanning, and cognitive assessment occurred from April 29, 2013, through February 14, 2017, when the children were aged 6 to 8 years. Details are provided in the eMethods in Supplement 1.

### MRI Acquisition and Analyses

**Figure 1.**
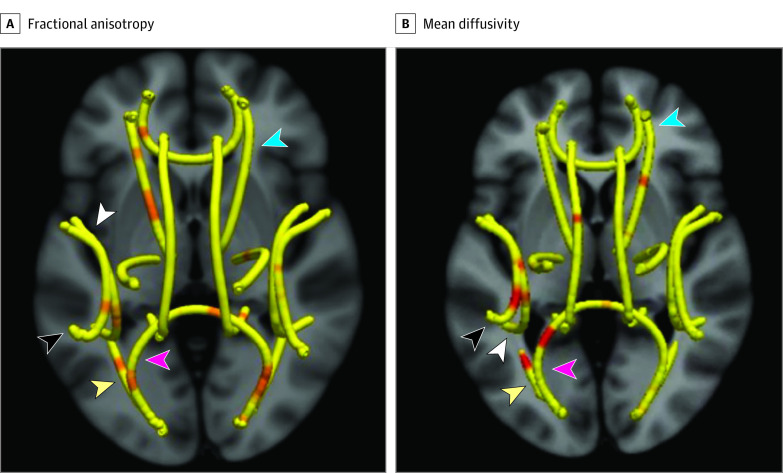
Tracts With Significantly Different Segments of Fractional Anisotropy or Mean Diffusivity Eighteen white matter tracts are observed. Segments highlighted in orange show significantly different mean fractional anisotropy or mean diffusivity values between children with postnatal growth failure (PGF) and those without PGF. The forceps major of the corpus callosum is indicated by a pink arrowhead, the right anterior thalamic radiation is indicated by a blue arrowhead, the left inferior longitudinal fasciculus is indicated by a yellow arrowhead, the left superior longitudinal fasciculus–parietal bundle is indicated by a black arrowhead, and the left superior longitudinal fasciculus–temporal bundle is indicated by a white arrowhead.

Structural, diffusion, and resting-state functional MRIs were acquired on a Siemens 3T Trio Tim scanner using a 24-channel phased-array head coil. Diffusion tensor imaging and resting-state functional MRI scans were obtained with the same scanner using an identical protocol for all participants during a single visit. The Tracts Constrained by Underlying Anatomy (TRACULA) tool within FreeSurfer 7.1^24^ was used for diffusion tensor imaging data processing and tractography to estimate the posterior probability of the 18 major white matter tracts. Among the 18 tracts, there were 5 tracts with segments that showed significant differences in both fractional anisotropy (FA) and mean diffusivity (MD) among the ELBW group (with or without PGF): the forceps major of the corpus callosum (Fmajor), right anterior thalamic radiation (RATR), left inferior longitudinal fasciculus, left superior longitudinal fasciculus–parietal bundle (LSLFP), and left superior longitudinal fasciculus–temporal bundle (Figure 1). The CONN toolbox^25^ was used for the seed-based functional connectivity analysis.^26^ To select the region of interest as a seed, the multivoxel–multivariate pattern analysis method was used, which showed group differences in 4 regions: the precuneus, the left and right superior lateral occipital cortex (sLOC), and the posterior cingulate cortex (PCC) (eFigure 2 in Supplement 1). A seed-based functional connectivity analysis was performed for whole-brain regions with the selected 4 regions of interest in the multivoxel–multivariate pattern analysis. Functional connectivity strength values were extracted from the brain regions of the preterm infants (uncorrected height threshold of *P* < .001 and cluster-level false discovery rate–corrected *P* < .05). The diffusion metrics of the selected tracts and the FCS values were used in the correlational analysis with clinical and neuropsychological measures. Details are provided in the eMethods in Supplement 1.

### Statistical Analysis

Image processing and statistical analyses were conducted through November 2021. Demographic and neurodevelopmental data were compared between the 3 groups. Data are expressed as numbers and percentages or as mean (SD) values. We conducted a 1-way analysis of variance and used a general linear model to examine the association of group differences in white matter microstructural properties, FCS, and cognitive outcomes. Partial correlations that controlled for birth weight were used to assess the strength of the association of white matter microstructural properties and FCS with cognitive outcomes among the combined groups of children born with ELBW and control participants. Statistical tests were 2-sided, and differences between the group correlations were considered significant at *P* < .05. All statistical analyses were performed using SPSS, version 24 software (IBM Corp).

## Results

A total of 82 MRI scans were assessed for 21 children born preterm with PGF (14 girls [66.7%] and 7 boys [33.3%]), 17 children born preterm without PGF (6 girls [35.3%] and 11 boys [64.7%]), and 44 children born full term (24 girls [54.5%] and 20 boys [45.5%]). The demographic characteristics of the included children are presented in eTable 1 in Supplement 1.

The children with PGF were born at a higher GA and with a lower birth weight than the children without PGF. The mean (SD) GA at birth was 27 weeks and 5 days (1 week and 6 days) for children with PGF and 26 weeks and 1 day (1 week and 1 day) for children without PGF. The mean (SD) birth weight was 801.1 (132.1) g for children with PGF and 830.7 (145.6) g for children without PGF. The morbidities associated with preterm birth are detailed in eTable 2 in Supplement 1. We found no significant differences in morbidities between the children with PGF and the children without PGF.

### Neurodevelopmental Outcomes

The mean (SD) values for the neurodevelopmental outcomes are shown in Table 1 and eTable 3 in Supplement 1. The mean (SD) Full-Scale Intelligence Quotient (FSIQ) score was significantly higher in the control group than in the group without PGF and the group with PGF (109.6 [16.1] vs 94.1 [15.8] vs 83.9 [17.0], respectively; *P* < .001). After adjusting for birth status, children with PGF had significantly lower FSIQ scores and 3 index scores of the subdomains of the Wechsler Intelligence Scale for Children (perceptual reasoning index, working memory index, and processing speed index) than did children without PGF. Some children were not able to complete the set of EF tests. The mean (SD) EF composite score was also significantly higher in the control group than in the group without PGF and the group with PGF (51.2 [7.1] vs 44.6 [8.7] vs 41.4 [8.0], respectively; *P* < .001). The mean differences in the EF composite scores between children with PGF and those without PGF were not statistically significant; however, after adjusting for GA at birth, children without PGF obtained higher EF composite scores than did children with PGF. The mean (SD) ATA score was higher for children with PGF (which is interpreted as worse function) than for children in the control group and children in the group without PGF (63.5 [9.4] vs 55.3 [9.8] vs 55.7 [8.0], respectively; *P* = .008). The mean differences in ATA scores between children with PGF and children without PGF were statistically significant, either with or without covarying for GA at birth. Therefore, the group with PGF had unfavorable attention function compared with the control group and the group without PGF.

**Table 1.** Neurodevelopmental Outcomes of the 3 Groups

Outcome	Mean (SD) value			*P* value^a^	*F*_2,79_	Partial η^2^	Mean difference (95% CI)		
	With PGF (n = 21)	Without PGF (n = 17)	Control group (n = 44)				Without PGF to control group	With PGF to control group	With PGF to without PGF
FSIQ score^b^	83.9 (17.0)	94.1 (15.8)	109.6 (16.1)	<.001	18.75	0.322	−14.71 (−26.10 to −3.32)^c^	−25.73 (−36.31 to −15.16)^d^	−11.03 (−24.03 to 1.98)
EF score	41.4 (8.0)^e^	44.6 (8.7)^f^	51.2 (7.1)^g^	<.001	10.58^h^	0.243	−6.58 (−12.26 to −0.90)^i^	−9.79 (−15.35 to −4.23)^d^	−3.21 (−9.79 to 3.37)
ATA score	63.5 (9.4)^j^	55.7 (8.0)	55.3 (9.8)	.008	5.19^k^	0.120	0.45 (−6.11 to 7.01)	8.23 (1.80 to 14.65)^c^	7.78 (0.01 to 15.55)^i^

Abbreviations: ATA, Advanced Test of Attention; EF, executive function; FSIQ, Full-Scale Intelligence Quotient; PGF, postnatal growth failure.

^a^ Calculated using general linear model (1-way analysis of variance) with post hoc Bonferroni test.

^b^ Measured using the Korean version of the Wechsler Intelligence Scale for Children, Fourth Edition, and the Korean Educational Development Institute–Wechsler Intelligence Scale for Children.

^c^ *P* < .01.

^d^ *P* < .001.

^e^ There were 17 children in the group with PGF.

^f^ There were 16 children in the group without PGF.

^g^ There were 36 children in the control group who completed the EF test.

^h^ *df* = (2,66).

^i^ *P* < .05.

^j^ There were 18 children in the group with PGF who completed the ATA test.

^k^ *df* = (2,76).

### Diffusion Tensor Imaging and FCS

**Figure 2.**
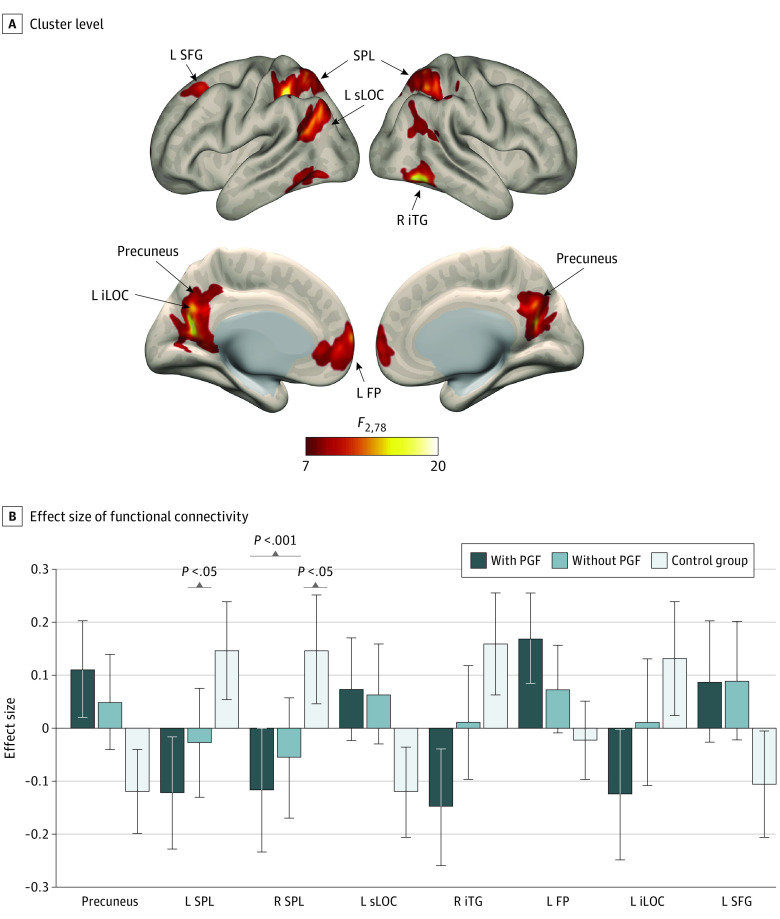
Differences in Functional Connectivity Between the 3 Groups From the Right Superior Lateral Occipital Cortex (sLOC) as a Seed Region A, Post hoc seed-to-voxel analysis using seed regions at the level of the right sLOC at the peak coordinates from multivoxel–multivariate pattern analysis. This analysis shows decreased functional connectivity between the sLOC and the bilateral superior parietal lobule (SPL) (red and yellow clusters) in children with postnatal growth failure (PGF). The color bar indicates the *F* statistic of between-group differences, with a threshold at *P* < .001 and false discovery rate correction (*P* < .05). B, Functional connectivity is measured from the sLOC as a seed to the following regions: precuneus, left SPL (L SPL), right SPL (R SPL), left sLOC (L sLOC), right inferior temporal gyrus (R iTG), left frontal pole (L FP), left inferior lateral occipital cortex (L iLOC), and left superior frontal gyrus (L SFG). Error bars indicate SDs.

In the analysis of the 3 groups, the general linear model revealed significant main associations in all 5 tracts. Post hoc comparison showed significantly lower mean (SD) FA in the group with PGF compared with the group without PGF in the Fmajor (0.498[0.067] vs 0.558 [0.044]) and left inferior longitudinal fasciculus (0.498[0.053] vs 0.529 [0.040]). In terms of MD, post hoc comparison showed significantly higher mean (SD) MD among children with PGF than among those without PGF in the LSLFP (8.312 [0.318] vs 7.902 [0.455]; originally calculated as milimeter squared per second and rescaled 10 000 times as MD × 10 000) (Table 2). However, analyses between preterm children adjusted for GA at birth showed significantly lower FA in the group with PGF than in the group without PGF in 4 tracts except for the RATR and significantly higher MD in the group with PGF than in the group without PGF in all 5 tracts (eTable 4 in Supplement 1). In the seed-based analyses, we found decreased FCS between the right sLOC and the right superior parietal lobule (SPL) in children with ELBW compared with children in the control group. In addition, after adjusting for GA, significantly decreased FCS was found in children with PGF compared with those without PGF between the precuneus and left superior frontal gyrus and between the right sLOC and the left SPL (Table 2 and Figure 2; eTable 4 in Supplement 1).

**Table 2.** General Linear Model of Diffusion Metrics and Functional Connectivity Strength of the 3 Groups

Metric	Main effects						Post hoc comparison, mean difference (95% CI)		
	Mean (SD) value			*P* value^a^	*F*_2,79_	Partial η^2^			
	With PGF (n = 21)	Without PGF (n = 17)	Control group (n = 44)				Without PGF to control group	With PGF to control group	With PGF to without PGF
Fmajor									
FA	0.498 (0.067)	0.558 (0.044)	0.570 (0.038)	<.001	16.110	0.290	−0.011 (−0.045 to 0.022)	−0.071 (−0.103 to −0.040)^b^	−0.060 (−0.099 to −0.022)^c^
MD^d^	9.243 (0.650)	8.709 (0.515)	8.828 (0.483)	.005	5.748	0.127	−0.119 (−0.494 to 0.256)	0.415 (0.067 to 0.763)^e^	0.534 (0.106 to 0.962)^e^
RATR									
FA	0.462 (0.120)	0.378 (0.078)	0.474 (0.107)	.007	5.251	0.117	−0.096 (−0.170 to 0.022)^c^	−0.012 (−0.080 to 0.056)	0.084 (0.0001 to 0.168)^e^
MD^d^	8.009 (0.652)	7.755 (0.326)	7.838 (0.287)	.04	3.436	0.080	NA	NA	NA
LILF									
FA	0.498 (0.053)	0.542 (0.034)	0.529 (0.040)	.005	5.711	0.126	0.013 (−0.016 to 0.043)	−0.031 (−0.058 to −0.003)^e^	−0.044 (−0.078 to −0.010)^c^
MD^d^	8.658 (0.387)	8.278 (0.371)	8.549 (0.485)	.03	3.692	0.085	NA	NA	NA
LSLFP									
FA	0.389 (0.050)	0.431 (0.045)	0.411 (0.044)	.02	3.951	0.091	NA	NA	NA
MD^d^	8.312 (0.318)	7.902 (0.455)	8.083 (0.393)	.007	5.359	0.119	−0.181 (−0.453 to 0.091)	0.229 (−0.024 to 0.482)	0.410 (0.010 to 0.721)^c^
LSLFT									
FA	0.382 (0.049)	0.421 (0.043)	0.390 (0.045)	.02	3.994	0.092	NA	NA	NA
MD^d^	8.402 (0.294)	8.070 (0.352)	8.176 (0.364)	.01	4.864	0.110	−0.106 (−0.347 to 0.135)	0.226 (0.002 to 0.450)^e^	0.332 (0.057 to 0.607)^e^
Seed or target									
Precuneus									
Left SFG	0.035 (0.156)	0.186 (0.165)	0.105 (0.174)	.03	3.818	0.088	NA	NA	NA
Left MidFG	0.074 (0.211)	0.193 (0.148)	0.142 (0.149)	.09	2.465	0.059	NA	NA	NA
AC	0.134 (0.202)	0.086 (0.111)	0.114 (0.201)	.73	0.314	0.008	NA	NA	NA
PC	0.605 (0.309)	0.639 (0.302)	0.680 (0.208)	.54	0.628	0.016	NA	NA	NA
PCC									
AC	0.055 (0.162)	0.142 (0.191)	0.249 (0.162)	<.001	9.998	0.202	−0.107 (−0.224 to 0.010)	−0.195 (−0.304 to −0.086)^b^	−0.088 (−0.047 to 0.222)
Right SPL	−0.075 (0.155)	−0.090 (0.188)	−0.062 (0.161)	.84	0.178	0.004	NA	NA	NA
Left SPL	−0.081 (0.144)	−0.112 (0.148)	−0.050 (0.159)	.36	0.105	0.026	NA	NA	NA
Right sLOC									
Right SPL	0.156 (0.273)	0.236 (0.278)	0.421 (0.241)	<.001	8.570	0.178	−0.185 (−0.364 to −0.005)^e^	−0.265 (−0.432 to −0.098)^b^	−0.080 (−0.285 to 0.125)
Left SPL	0.152 (0.267)	0.286 (0.209)	0.425 (0.220)	<.001	10.337	0.207	−0.139 (−0.300 to 0.022)	−0.273 (−0.423 to −0.124)^b^	−0.135 (−0.319 to 0.049)
Left sLOC									
Right SPL	0.127 (0.286)	0.231 (0.276)	0.413 (0.251)	<.001	9.024	0.186	−0.182 (−0.368 to 0.003)	−0.286 (−0.458 to −0.114)^b^	−0.104 (−0.316 to 0.108)
Left SPL	0.271 (0.316)	0.385 (0.277)	0.569 (0.240)	<.001	9.607	0.196	−0.184 (−0.370 to 0.002)	−0.298 (−0.471 to −0.125)^b^	−0.114 (−0.327 to 0.099)

Abbreviations: AC, anterior division of the cingulate gyrus; FA, fractional anisotropy; Fmajor, forceps major of the corpus callosum; LILF, left inferior longitudinal fasciculus; LSLFP, left superior longitudinal fasciculus–parietal bundle; LSLFT, left superior longitudinal fasciculus–temporal bundle; MD, mean diffusivity; MidFG, middle frontal gyrus; NA, not applicable; PC, posterior division of the cingulate gyrus; PCC, posterior cingulate cortex; PGF, postnatal growth failure; RATR, right anterior thalamic radiation; SFG, superior frontal gyrus; sLOC, superior lateral occipital cortex; SPL, superior parietal lobule.

^a^ Calculated using general linear model (1-way analysis of variance) with post hoc Bonferroni test.

^b^ *P* < .001.

^c^ *P* < .01.

^d^ Originally calculated as millimeter squared per second and rescaled 10 000 times as MD × 10 000.

^e^ *P* < .05.

### Correlations With Neurodevelopmental Outcomes

Among all of the participants, the ATA score was negatively correlated with the FA of the left inferior longitudinal fasciculus (*r* = −0.238; *P* = .04) and positively correlated with the MD of the Fmajor (*r* = 0.225; *P* = .047) after adjusting for birth weight (Table 3). The negative correlation between the FA of the Fmajor and the ATA score did not reach significance (*r* = −0.207; *P* = .07). The FSIQ was positively correlated with the FCS between the left sLOC and the right SPL (*r* = 0.262; *P* = .02) and the left sLOC and the left SPL (*r* = 0.286; *P* = .01). The EF composite score was positively correlated with the FCS between the left sLOC and the right SPL (*r* = 0.367; *P* = .002) and between the left sLOC and the left SPL (*r* = 0.324; *P* = .007). Concurrently, the ATA score was positively correlated with the FCS between the precuneus and anterior division of the cingulate gyrus (*r* = 0.225; *P* = .048). However, it was negatively correlated with the FCS between the PCC and the right SPL (*r* = −0.269; *P* = .02) and between the PCC and the left SPL (*r* = −0.338; *P* = .002).

**Table 3.** Partial Correlation Coefficients Between Fractional Anisotropy, Mean Diffusivity, or Functional Connectivity Strength and Neurodevelopmental Outcomes in the Combined Sample of Children Born Full Term and Children Born Preterm, Controlling for Birth Weight^a^

Partial correlation	Full-Scale Intelligence Quotient^b^		Executive function		Advanced Test of Attention	
	*r* Value	*P* value	*r* Value	*P* value	*r* Value	*P* value
**Diffusion metric**						
Fractional anisotropy						
Fmajor	0.138	.22	0.199	.10	−0.207	.07
RATR	0.022	.84	0.121	.32	−0.111	.33
LILF	0.016	.88	0.102	.41	−0.238	.04
LSLFP	0.013	.91	0.230	.06	−0.021	.86
LSLFT	−0.023	.84	0.119	.33	−0.019	.87
Mean diffusivity						
Fmajor	−0.088	.43	−0.101	.41	0.225	.047
RATR	0.036	.75	−0.078	.53	0.181	.11
LILF	0.044	.70	−0.081	.51	0.105	.36
LSLFP	−0.016	.89	−0.235	.05	0.160	.16
LSLFT	0.015	.90	−0.131	.29	0.158	.17
Functional connectivity strength						
Seed or target						
Precuneus						
Left SFG	0.053	.64	−0.069	.58	0.112	.33
Left MidFG	0.188	.09	0.091	.49	−0.199	.08
AC	−0.114	.31	−0.152	.22	0.225	.048
PC	0.201	.07	0.072	.56	−0.098	.39
PCC						
AC	0.152	.18	−0.088	.48	−0.048	.68
Right SPL	−0.028	.81	0.004	.98	−0.269	.02
Left SPL	0.046	.68	0.125	.31	−0.338	.002
Right sLOC						
Right SPL	0.156	.17	0.184	.13	−0.064	.58
Left SPL	0.119	.29	0.147	.23	−0.142	.22
Left sLOC						
Right SPL	0.262	.02	0.367	.002	0.069	.55
Left SPL	0.286	.01	0.324	.007	−0.024	.84

Abbreviations: AC, anterior division of the cingulate gyrus; Fmajor, forceps major of corpus callosum; LILF, left inferior longitudinal fasciculus; LSLFP, left superior longitudinal fasciculus–parietal bundle; LSLFT, left superior longitudinal fasciculus–temporal bundle; MidFG, middle frontal gyrus; PC, posterior division of the cingulate gyrus; PCC, posterior cingulate cortex; RATR, right anterior thalamic radiation; SFG, superior frontal gyrus; sLOC, superior lateral occipital cortex; SPL, superior parietal lobule.

^a^ Partial correlation coefficients with controlling for birth weight.

^b^ Measured using the Korean version of the Wechsler Intelligence Scale for Children, Fourth Edition, and the Korean Educational Development Institute–Wechsler Intelligence Scale for Children.

## Discussion

To our knowledge, this is the first study to simultaneously disclose structural and functional alterations in the brains of early school–aged children born preterm in association with postnatal growth patterns. Decrements in EF are prevalent among preterm children.^27,28,29^ Furthermore, poor postnatal growth may be associated with neurodevelopmental outcomes in infancy and early adulthood.^30,31,32^ Social factors are associated with cognitive outcome variations among children born preterm.^33^ In line with previous studies, we observed that the FSIQ and EF composite scores were lower among children with PGF and those without PGF than among children born full term in the control group. These findings suggest that very preterm birth may have an adverse effect on the developmental catch-up of intelligence and EF until early school age, which is consistent with previous studies.^34,35^ Furthermore, children with PGF had worse attention function than did those without PGF or children in the control group. This finding indicates that very preterm birth with PGF has a negative association with attention ability, which persists until early school age. However, children without PGF were able to catch up to equally comparable development in attention ability until early school age. This result is similar to a nationwide Japanese study demonstrating that preterm infants born small for gestational age (SGA) with poor postnatal growth were at risk of attention problems until school age.^36^ These findings can be interpreted as attention function possibly being more sensitive than cognitive functions, including EF, to postnatal growth.

At 24 weeks of gestation, significant refinement of neural connections occurs^37^; axons develop in the white matter and make connections, neurons migrate, and preoligodendrocytes mature into myelin-producing mature oligodendrocytes.^38^ Therefore, exposure to growth restriction during this critical period results in definite alterations of the brain. The observed white matter changes, along with decreased FA and increased MD, in children with PGF could be interpreted as decreased levels of myelination, as reported in animal models of preterm births,^39^ which suggests delayed maturation. These microstructural alterations were also observed in several previous studies, including a systematic review showing that preterm neonates displayed decreased FA and increased MD on MRI scans at term-equivalent age.^40,41^ Some studies have shown that the brain microstructure can be improved through environmental interventions.^42,43^ Cho et al^44^ found that the differences disappeared by school age and stated that white matter microstructure in preterm infants caught up with that of term-born controls by school age. In contrast, we were unable to detect delayed white matter maturation patterns showing decreased FA and increased MD in children without PGF as observed in children with PGF. Therefore, we suggest that the catch-up development of white matter microstructure could occur by early school age, especially for children without PGF.

The development of thalamic radiation requires a long time from the late embryonic period to the third trimester of gestation.^45^ Therefore, the RATR showed a different pattern than the other 4 tracts; for children with PGF, regions of increased FA in the segment of the RATR might be associated with a late compensatory effect to early injury during development.^46,47^ Axonal projections contained within the RATR are known to connect the thalamus, striatum, and frontal lobes and have been implicated in higher-order cognitive functions.^48^ Injury to or dysmaturity of fiber connections of the thalamic radiations in preterm children may account, in part, for individual variations in behavioral abilities thought to involve functions of the frontal lobes, including attention.^49,50,51^

Microstructural alterations and functional connectivity are not necessarily identical.^52,53^ The FCS between the SPL and the sLOC showed definite group differences; however, we were unable to observe differences through diffusion tensor imaging analysis in the same region. The SPL is a subregion of the dorsal posterior parietal cortex, which has diverse convergent functional architecture.^54^ The SPL is associated with selective attention^55^ and may play a role in shifting attention.^56^ As mentioned previously, children with PGF had worse attention function than did children without PGF and children in the control group, and the SPL might be a vulnerable cortical lesion in preterm children with PGF. Although not statistically significant, similar to previous studies, a decreased resting-state FCS was found in children with PGF compared with children without PGF or children in the control group in most connections. The Fmajor, in which an altered microstructure has been found in children with PGF, is the posterior portion of the corpus callosum connecting the 2 occipital lobes at the back of the brain.^57^ In regard to attention function, lower FA or higher MD of the Fmajor was associated with worse attention function, although not statistically significant in terms of FA. This pattern of discovery is generally consistent with the findings of previous studies of the corpus callosum.^58,59^

The superior longitudinal fasciculus–parietal bundle connects the SPL to the ipsilateral frontal areas and is involved in spatial attention. Spatial attention is important for higher-order cognitive functions and is closely associated with EF.^60^ The LSLFP, where increased MD was observed in children with PGF compared with those without PGF, showed a negative but not statistically significant correlation with EF function. Since the SPL is also a region where differences in the FCS among children born with ELBW were confirmed in this study, differences in microstructure may also be associated with functional connectivity.

Specific areas in the brain demonstrated group differences in microstructure properties or FCSs and properties in which significant structure-behavior or function-behavior associations did not match. These findings suggest that individual differences in cognitive function may depend on multiple pathways and tissue microstructure properties beyond those found to differ between preterm and full-term groups, such as myelin content and fiber, as well as axonal properties. In addition, the organization of functional connections may be associated with many socioenvironmental factors as well as with neurobiological factors.

### Limitations

Although this study provides insight into the brain maturation and cognitive abilities of children born preterm at early school age from the novel perspective of postnatal growth, several limitations must be considered. First, we excluded infants born SGA from the analyses, and the perinatal risk factors between children with and children without PGF showed no differences, but PGF was defined only by weight at hospital discharge regardless of birth weight. Second, despite the small sample size of previous studies of individuals born preterm,^21,22,44,61,62^ the sample size of our study was modest. The limited sample size may have restricted the identification of subtle group differences in functional connectivity and group-specific associations between the FCS and cognitive abilities. Third, the 2 tests were highly correlated, but children born full term from another SNUCH cohort completed a different version of the Wechsler Intelligence Scale for Children. Fourth, the development of early school–aged children was decided by environmental factors. We considered the Hollingshead Four Factor Index of Social Status, which did not differ between groups, but there could be more meaningful social factors that were not included. These factors may limit the generalizability of the current findings.

## Conclusions

This study suggests a long-lasting association of very preterm birth and poor postnatal growth with cognitive ability, organization of brain microstructure, and functional brain networks in early school–aged children. Although the precise neuronal mechanisms remain obscure, preterm birth with poor growth might be associated with long-term brain maturation and development; structurally, the Fmajor and, functionally, the SPL may be the most vulnerable regions. Appropriate growth during this critical period is important for long-lasting structural and functional brain maturation and neurodevelopment in preterm infants. However, PGF is associated with nutritional and nonnutritional factors; nutritional intervention alone would not achieve optimal brain connectivity. A further comprehensive investigation of early neonatal growth and white matter tracts would contribute to our understanding of brain-behavior associations in the context of preterm birth.

## Notes

Supplement 1.
eMethods.

eReferences.
**eFigure 1.** Flowchart Demonstrating the Selection of Study Participants From the Enrolled Children **eFigure 2.** Selection of Seed ROIs Using MVPA **eTable 1.** Participant Characteristics by Group With Subgroup Comparisons **eTable 2.** Perinatal Risk Factors for Preterm Infants **eTable 3.** Neurodevelopmental Outcomes in Preterm Groups, Adjusted for Gestational Age at Birth **eTable 4.** Diffusion Metrics and Functional Connectivity Strength for Preterm-Born Children With or Without PGF, Adjusted for Gestational Age at Birth

Supplement 2.
Data Sharing Statement
