# Supplementary material for: Association of Brain Microstructure and Functional Connectivity With Cognitive Outcomes and Postnatal Growth Among Early School–Aged Children Born With Extremely Low Birth Weight
Source: JAMA Netw Open. 2023 Mar 2;6(3):e230198. doi: 10.1001/jamanetworkopen.2023.0198 (PMC9982697; doi:10.1001/jamanetworkopen.2023.0198)
Supplement: Supplement 1. — eMethods. eReferences. eFigure 1. Flowchart Demonstrating the Selection of Study Participants From the Enrolled Children eFigure 2. Selection of Seed ROIs Using MVPA eTable 1. Participant Characteristics by Group With Subgroup Comparisons eTable 2. Perinatal Risk Factors for Preterm Infants eTable 3. Neurodevelopmental Outcomes in Preterm Groups, Adjusted for Gestational Age at Birth eTable 4. Diffusion Metrics and Functional Connectivity Strength for Preterm-Born Children With or Without PGF, Adjusted for Gestational Age at Birth [file jamanetwopen-e230198-s001.pdf]

Supplementary Online Content

Kim SY, Kim EK, Song H, et al. Association of brain microstructure and functional connectivity with cognitive outcomes and postnatal growth among early school–aged children born with extremely low birth weight. *JAMA Netw Open.* 2023;6(3):e230198. doi:10.1001/jamanetworkopen.2023.0198

eMethods.
eReferences.
eFigure 1. Flowchart Demonstrating the Selection of Study Participants from the Enrolled Children
eFigure 2. Selection of Seed ROIs Using MVPA
eTable 1. Participant Characteristics by Group With Subgroup Comparisons
eTable 2. Perinatal Risk Factors for Preterm Infants
eTable 3. Neurodevelopmental Outcomes in Preterm Groups, Adjusted for Gestational Age at Birth
eTable 4. Diffusion Metrics and Functional Connectivity Strength for Preterm-Born Children With or Without PGF, Adjusted for Gestational Age at Birth

This supplementary material has been provided by the authors to give readers additional information about their work.

eMethods.

Study population

The Seoul National University Children's Hospital (SNUCH) preterm follow-up cohort was used to evaluate the associations of cognitive ability, executive function (EF), and behavioral characteristics with brain connectivity (http://clinicaltrials.gov/show/NCT02741934). Children who were born between 2008 and 2009 and admitted to the SNUCH neonatal intensive care unit (NICU) were recruited. The SNUCH attention deficit hyperactivity disorder (ADHD) cohort was used to identify the comprehensive pathophysiology of ADHD (http://clinicaltrials.gov/show/NCT02623114). From the SNUCH preterm follow-up cohort, we included extremely low birth weight (ELBW) children (birth weight <1000 g or gestational age [GA] <32 completed weeks), and typically developing children born at full term (GA ≥37 weeks and birth weight ≥2500 g) for the control group. Additionally, from the SNUCH ADHD cohort, we included age-matched typically developing children born at full term children who served as control group. We defined severe brain injury as intraventricular hemorrhage (IVH) exceeding grade 3 or periventricular leukomalacia. Children whose birth weight was <3rd percentile or who had severe brain injury were excluded. In the ELBW group, postnatal growth failure (PGF) was defined as a weight <3rd percentile for postmenstrual age based on the Fenton growth chart at discharge from the NICU.

Finally, 98 parents gave consent for their children to participate in the study. However, 2 preterm children failed to complete magnetic resonance imaging (MRI) and 12 children were excluded from the analyses because of birth weight or a paucity of birth data. After MRI preprocessing, the images of 2 children could not be analyzed and were therefore excluded. Finally, 82 MRIs were assessed: those of children with PGF [N=21], those of children without PGF [N=17], and those of the control group (N=44).

Magnetic resonance imaging acquisition

Due to poor cooperation from infants because of their young age, 13 patients (5 from the control group and 8 from the ELBW group) underwent MRI after administering chloral hydrate syrup. All imaging data were acquired using a Siemens 3T Trio Tim scanner (Berlin, Germany) with a 24-channel phased-array head coil. Structural brain data were acquired using a 3D T1-weighted magnetization-prepared rapid gradient echo (MPRAGE) sequence (repetition time = 1900 ms, echo time = 3.1 ms, flip angle = 9, field of view = 256 mm, 176 sagittal slices, and 1 mm isotropic resolution). Resting-state functional MRI (rs-fMRI) data were acquired using an interleaved gradient echo planer T2*-weighted sequence (repetition time = 3000 ms, echo time = 40 ms, flip angle = 90, field of view = 240 mm, slice thickness = 4 mm, matrix size = 64 × 64, voxel resolution = 3.75 mm × 3.75 mm × 4 mm, 190 volumes scan [9 min, 30 s]). Diffusion tensor imaging (DTI) data were acquired in 30 directions with a b-value of 700 (repetition time = 1000 ms, echo time = 88 ms, flip angle = 90, matrix size = 128 × 128, voxel resolution = 1.875 mm × 1.875 mm × 3.5 mm, 50 axial slices).

Diffusion MRI measures and analyses

Structural images were processed using a fully automated processing pipeline for volumetric segmentation and cortical thickness estimation within FreeSurfer 7.1 (https://freesurfer.net). Briefly, this procedure includes skull stripping, bias correction for inhomogeneity field correction, Talairach transformation, intensity normalization, subcortical segmentation of gray or white matter, and tessellation of the gray/white matter boundary and pial surface using continuity information and image intensities from the structural volume.[1] The processed structural data and information were used to register the DTI data. The TRActs Constrained by UnderLying Anatomy (TRACULA) tool within FreeSurfer 7.1 was used for DTI data processing and tract segmentation. TRACULA is a processing pipeline that performs global probabilistic tractography to estimate the posterior probability of the following 18 major white matter tracts: the corticospinal tract, cingulum–cingulate gyrus bundle, cingulum angular bundle, inferior longitudinal fasciculus (ILF), superior longitudinal fasciculus-temporal bundle (SLFT), superior longitudinal fasciculus-parietal bundle (SLFP), anterior thalamic radiation (ATR), and uncinate fasciculus in each hemisphere; and the forceps major (Fmajor) and minor of the corpus callosum. The preprocessing stage was performed as follows: eddy current and head movement correction, and intra-subject and inter-subject registration to the FreeSurfer common template space by the bbregister program of FreeSurfer. Tensor fitting was also applied for least-squares tensor estimation using FMRIB Software Library (FSL; https://fsl.fmrib.ox.ac.uk/fsl/fslwiki). TRACULA invoked the bedpost tool of FSL to apply the ball-and-stick model of diffusion to the DTI data,[2] and estimated the posterior probability of each pathway in participants with a pathway for prior information on each position of the tract combined with the participant's anatomical segmentation labels. Finally, TRACULA combined diffusion measures (FA, axial diffusivity, mean diffusivity [MD], and radial diffusivity) along each pathway and extracted values from each diffusion measure of each

© 2023 Kim SY et al. *JAMA Network Open.*

pathway. Tracts with segments that showed significantly different diffusion measures were identified among the ELBW group. We selected 5 tracts that included important segments that showed significant differences in both FA and MD: Fmajor, right ATR (RATR), left ILF (LILF), left SLFP (LSLFP), and left SLFT (LSLFT) (Figure 1). The diffusion measures from the selected tracts were used to investigate their relationship with clinical measures.

Functional connectivity analyses

The CONN toolbox (https://web.conn-toolbox.org) was used to select the region of interest (ROI) as a seed using multivoxel/multivariate pattern analysis (MVPA) and for seed-based functional connectivity analysis.[3] Functional data were preprocessed for realignment and slice timing, co-registered with structural data, segmented, normalized to the pediatric Montreal Neurological Institute template of 7–11 year olds[4] for 2 mm iso-voxel resolution, and a Gaussian kernel of 8 mm full-width at half maximum was applied for smoothing. Linear motion parameters (X, Y, and Z-axes) and rotational motion parameters (pitch, roll, and yaw) were estimated from the realignment processing. The means for head motion were not significantly different between groups. The estimated motion parameters were applied as the first-level covariates. The artifact detection tool within the CONN toolbox was used to identify outliers satisfying at least 1 of the following criteria: global signal threshold $Z \geq 3.0$, absolute subject motion threshold ≥0.5 mm, absolute subject rotation threshold ≥0.05 radians, or scan-to-scan motion threshold ≥0.2 radians. The detected outliers were used as additional covariates. A low-pass filter (0.008–0.09 Hz) was applied to isolate low-frequency fluctuations for resting state data.

The MVPA was conducted for the entire multivariate pattern of pairwise connections between all regions in the brain[3] in the children born preterm with and without PGF, and the control group. MVPA was performed to identify seed ROIs for use in functional connectivity analysis. The MVPA reduced the dimensionality of the multivoxel pattern using the principal component analysis, and the effects of age and sex were controlled as covariates. Seed ROIs were selected by running 5000 permutations to reduce Type I errors, then a height-level threshold of $p < .001$ was set and the cluster-level false discovery rate (FDR) was corrected to $p < .05$. The functional connectivity analysis was performed using the source ROIs from the MVPA. The MVPA showed group differences with regards to whole brain functional connectivity patterns in 4 regions: the precuneus, left and right superior lateral occipital cortex, and posterior cingulate cortex (eFigure 2).

The functional connectivity differences among the three groups (children born preterm with and without PGF, and the control group) were tested with an uncorrected height threshold of $p < .001$ and cluster-level FDR corrected $p < .05$. For all second-level analyses, the connectivity values were calculated and extracted using Fisher's Z-transformed values. The extracted connectivity strength values were then used in the correlational analysis with clinical and psychological measures.

Cognitive assessment

The participants completed a battery of standardized measures of cognitive skills, EF, attention ability, and behavioral assessment. Psychologists blinded to the perinatal findings or post-discharge details assessed the children. General intellectual ability was assessed using the Korean version of the Wechsler Intelligence Scale for Children, Fourth Edition (K-WISC-IV).[5] Children of the control group from the SNUCH ADHD cohort were evaluated for intelligence using the Korean Educational Development Institute-Wechsler Intelligence Scale for Children (KEDI-WISC).[6] Participants completed the Advanced Test of Attention (ATA), Children's Color Trails Test (CCTT), STROOP Color and Word Test (STROOP), and Wisconsin Card Sorting Test (WCST) to evaluate EF.

After testing using the K-WISC-IV or KEDI-WISC, the Full-Scale Intelligence Quotient was used to assess general intelligence. The index scores of the 4 subdomains of the WISC-IV were evaluated for more specific cognitive information in ELBW children. The verbal comprehension index was used as a measure of knowledge of word meanings and verbal reasoning abilities; the perceptual reasoning index, as a measure of visuoconstructional skills and visual reasoning abilities; the working memory index, as a measure of immediate and working memory; and the processing speed index, as a measure of speed and accuracy of information processing. Each index score was standardized by age, with a mean of 100 and a standard deviation (SD) of 15.

The ATA is a computerized cognitive test that measures a child or adolescent's sustained and selective attention and impulsivity.[7] The ATA shows the age-adjusted T-scores (mean=50, SD=10) of 4 indices: omission errors, commission errors, response time, and the SD of overall response times (throughout the task). In the current study, we defined the ATA score as the average of 8 T-scores. A high T-score indicated inattention or impulsivity.

The CCTT was designed to provide an objectively-scored measure of sustained visual attention, sequencing, psychomotor speed, and cognitive flexibility.[8] The CCTT comprises 2 parts (CCTT1 and CCTT2);

© 2023 Kim SY et al. *JAMA Network Open.*

CCTT1 requires children to quickly and correctly sequence numbers with 2 different background colors between odd and even numbers, whereas CCTT2 requires the children to connect circles in ascending order while alternating between 2 different background colors. The score reflects the time from the initiation to completion of the task. Difference interference was calculated as the difference between the raw task completion scores of CCTT1 and CCTT2. Data are presented as T-scores (M=50, SD=10), adjusted for age and sex. In this study, the CCTT score was defined as the average of the CCTT1 and CCTT2 T-scores.

The STROOP test was developed for children aged 5–14 years and has been widely applied to assess cognitive inhibition, or the ability to ignore the interference from irrelevant stimuli, which mainly reflects frontal lobe function;[9] the Korean version has been standardized.[10] The STROOP test comprises 3 card sessions: word, color, and incongruent color-word. Each of the 100 cards contains a stimulus, and the children must read them aloud as quickly as possible. The total time spent reading the cards is checked during the test. Interference control (inhibition of habitual response) was measured in this test, and the T-score for age was registered for data analysis. In this study, the STROOP score was defined as the average of 3 T-scores.

The computerized WCST was used in the current study, which requires the development and maintenance of precise problem-solving strategies under various test conditions, and cognitive flexibility.[11] The program calculates T-scores for perseverative responses, perseverative errors, nonperseverative errors, conceptual level responses, and raw scores for the number of categories completed. In this study, the T-score of the number of categories completed (M=50, SD=10) was calculated manually. The WCST score was defined as the average of 5 T-scores: perseverative responses, perseverative errors, nonperseverative errors, conceptual level responses, and number of categories completed. Ultimately, we defined the EF composite score as the average of the CCTT, STROOP, and WCST scores. Higher scores are indicative of better test performance.

Statistical analyses

Demographic variables

We computed separate one-way analyses of variance (ANOVA) to compare continuous variable demographics, including GA, birth weight, age at scan, and socioeconomic status, between groups with the Bonferroni post hoc test to follow up on significant differences, depending on the homogeneity of variance. Fisher's exact test was used to compare categorical variables across the 3 groups (children born preterm with and without PGF, and the control group).

Group comparisons of white matter microstructural properties and cognitive measures

We conducted a one-way ANOVA, and a general linear model was computed to examine the contribution of the group (children born preterm with and without PGF, and the control group) to white matter microstructural properties, functional connectivity strength (FCS), and cognitive outcomes. Two additional series of general linear models, including only ELBW children with GA as a covariate, were conducted to examine the contribution of the group (children born preterm with and without PGF) to white matter microstructural properties, FCS, and cognitive outcomes, independent of GA. Differences between the groups were considered significant at $p<.05$.

Correlations between white matter microstructural or FCS and cognitive measures

Partial correlations controlling for birth weight were used to assess the strength of the association between white matter microstructural properties and cognitive outcome, and between FCS and cognitive outcome in the combined groups of ELBW and control participants. Correlations were considered significant at $p<.05$. All statistical analyses were performed using SPSS version 24 (IBM Corp, Armonk, NY).

© 2023 Kim SY et al. *JAMA Network Open.*

eReferences.

1. Fischl B, Van Der Kouwe A, Destrieux C, et al. Automatically parcellating the human cerebral cortex. Cerebral cortex 2004;14:11-22.
2. Behrens TE, Berg HJ, Jbabdi S, Rushworth MF, Woolrich MW. Probabilistic diffusion tractography with multiple fibre orientations: What can we gain? Neuroimage 2007;34:144-55.
3. Whitfield-Gabrieli S, Ford JM. Default mode network activity and connectivity in psychopathology. Annual review of clinical psychology 2012;8:49-76.
4. Fonov V, Evans AC, Botteron K, et al. Unbiased average age-appropriate atlases for pediatric studies. Neuroimage 2011;54:313-27.
5. Goo M, Oh S, Lee S, Paik Y, Lee J, Hwang K. Korean-Wechsler Intelligence Scale for Children-IV (K-WISC-IV). Seoul: Hakjisa 2016.
6. Kim M, Kim Z. A study on the abbreviated form of the K-WISC. Seoul J Psychiatr 1986;11:194-201.
7. Shin M-S, Choi H, Kim H, Hwang J-W, Kim B-N, Cho S-C. A study of neuropsychological deficit in children with obsessive-compulsive disorder. European Psychiatry 2008;23:512-20.
8. Shin M, Koo H. Children's Color Trails Test. Seoul: Hakjisa 2007.
9. Golden CJ, Freshwater SM. Stroop color and word test. 1978.
10. Shin M, Park M. STROOP: Color and Word Test Children's Version for Ages 5-14. Seoul: Hakjisa 2007.
11. Morin CM. Insomnia: Psychological assessment and management: Guilford press; 1993.
12. Lee H-L, Oh K-J, Hong K-E, Ha E-H. Clinical validity study of Korean CBCL through item analysis. Journal of the Korean Academy of Child and Adolescent Psychiatry 1991;2:138-49.
13. Han DH, Woo J, Jeong JH, Hwang S, Chung U-S. The Korean version of the pediatric symptom checklist: psychometric properties in Korean school-aged children. Journal of Korean medical science 2015;30:1167-74.

© 2023 Kim SY et al. *JAMA Network Open.*

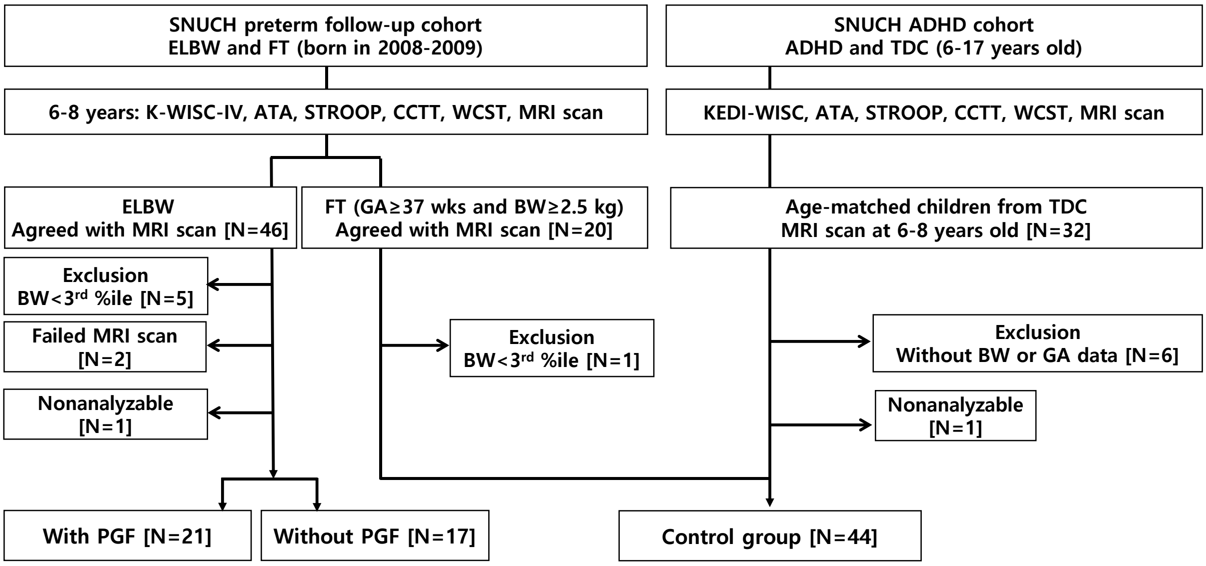

eFigure 1. Flowchart Demonstrating the Selection of Study Participants from the Enrolled Children

ADHD, attention deficit hyperactivity disorder; ATA, Advanced Test of Attention; BW, birth weight; CCTT, Children's Color Trails Test; ELBW, extremely low birth weight; FT, full term; GA, gestational age; K-WISC-IV, Korean version of the Wechsler Intelligence Scale for Children, Fourth Edition; KEDI-WISC, Korean Educational Development Institute-Wechsler Intelligence Scale for Children; MRI, magnetic resonance imaging; PGF, postnatal growth failure; SNUCH, Seoul National University Children's Hospital; STROOP, STROOP Color and Word Test; TDC, typically developing children; WCST, Wisconsin Card Sorting Test

© 2023 Kim SY et al. *JAMA Network Open.*

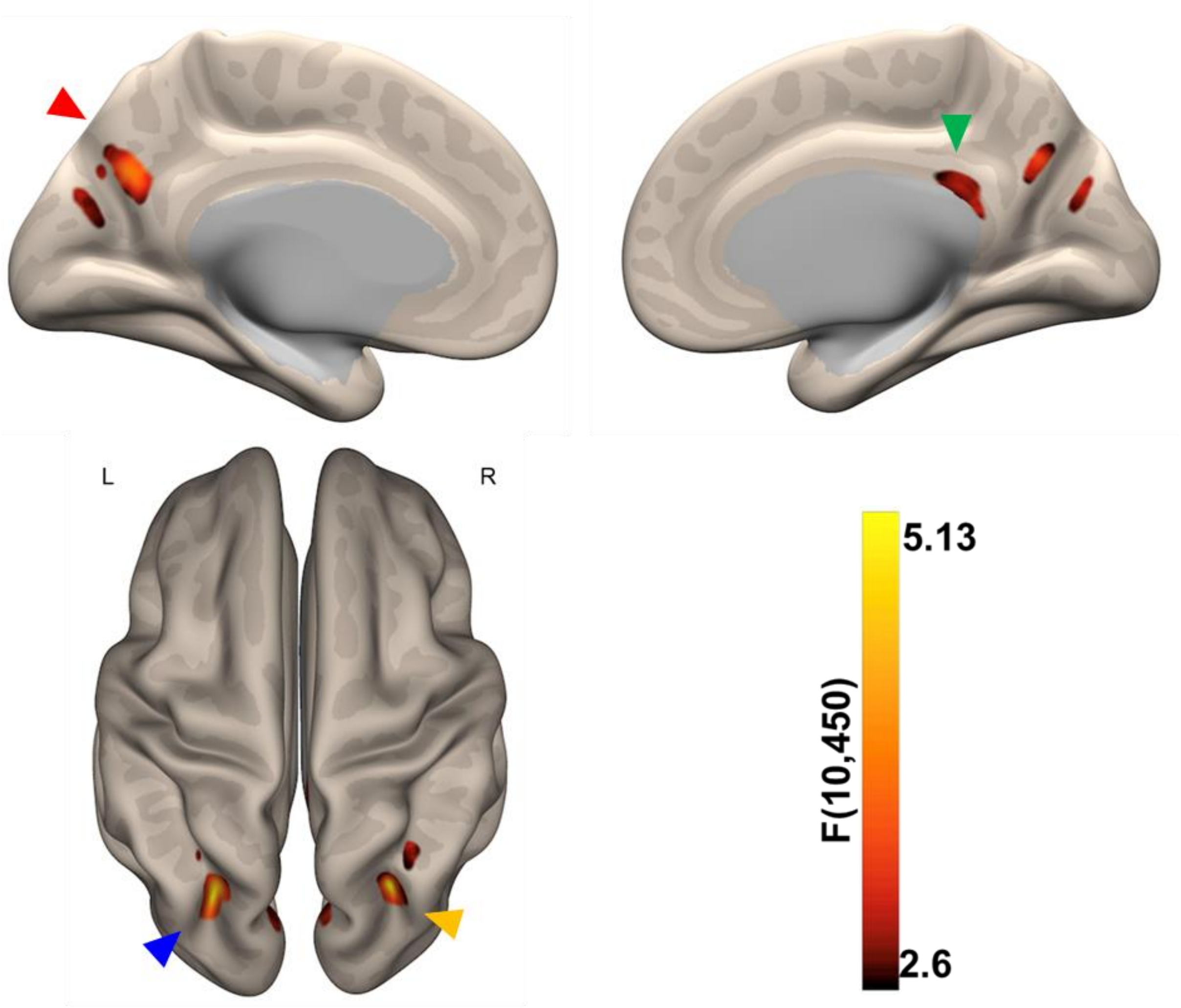

eFigure 2. Selection of Seed ROIs Using MVPA

Differences in brain connectivity profile indexed at a voxel level using MVPA. MVPA results are overlaid on a representative semi-inflated brain template. Yellow-orange clusters indicate brain regions that show different patterns of functional connectivity between the children born preterm with PGF and the typically developing children born at full term groups. The color bar indicates the F-statistic of between-group differences. Four clusters were identified: Precuneus is annotated by a red arrow, left superior occipital cortex is annotated by a blue arrow, right superior occipital cortex is annotated by an orange arrow, and posterior cingulate cortex is annotated by a green arrow. On the F-maps, a voxel-wise threshold of $p<0.001$ was used. Cluster-level FWE correction was applied at $p<0.05$.

FWE, family-wise error; MVPA, multivoxel/multivariate pattern analysis; PGF, postnatal growth failure; ROI, region of interest

© 2023 Kim SY et al. *JAMA Network Open.*

eTable 1. Participant Characteristics by Group With Subgroup Comparisons

	With PGF [N=21]	Without PGF [N=17]	Control group [N=44]	*P*	Post hoc Comparison		
					Without PGF to control group	With PGF to control group	With PGF to Without PGF
GA, weeks	27^{+5} (1^{+6})	26^{+1} (1^{+1})	>37^{+0}	<.001	<.001	<.001	0.001
Birthweight, grams	801.05 (132.1)	830.7 (145.6)	3265.7 (405.2)	<.001	<.001	<.001	>.99
Male	7/21 (33.3%)	11/17 (64.7%)	20/44 (45.5%)	.15			
Female	14/21 (66.7%)	6/17 (35.3%)	24/44 (54.5%)				
SES	49.3 (7.3)	44.4 (4.2)	47.6 (9.8)	.19	>.99	0.97	0.79
Age at	7.0 (0.4)	7.0 (0.4)	7.0 (0.6)	.48	0.55	>.99	0.22

Values are presented as means (standard deviations) or frequencies (percentage), as appropriate.
*P-values are calculated using one-way analysis of variance and the chi-square test.
SES was measured using the Hollingshead Four-Factor Index of Social Status.
GA, gestational age at birth; PGF postnatal growth failure; SES, socioeconomic status

© 2023 Kim SY et al. *JAMA Network Open.*

eTable 2. Perinatal Risk Factors for Preterm Infants

	With PGF [N=21]	Without PGF [N=17]	*P*
GA, weeks	27^{+5} (1^{+6})	26^{+1} (1^{+1})	.008
Birth weight, grams	801.05 (132.1)	830.7 (145.6)	.51
Male	7/21 (33.3%)	11/17 (64.7%)	.05
Female	14/21 (66.7%)	6/17 (35.3%)	
HCAM	2/21 (9.5%)	6/17 (35.3%)	.11
C/S	18/21 (85.7%)	13/17 (76.5%)	.68
PDA	19/21 (90.5%)	16/17 (94.1%)	>.99
Culture-proven sepsis	10/21 (47.6%)	4/17 (23.5%)	.13
Moderate or severe BPD	12/21 (57.1%)	6/17 (35.3%)	.21
NEC	4/21 (19.0%)	0/17 (0.0%)	.11
ROP ≥ grade 3 or laser	5/21 (23.8%)	4/17 (23.5%)	>.99
Brain injuries			
cranial USG, IVH≥2	3/21 (14.3%)	1/17 (5.9%)	.44
abnormal term MRI	1/10	1/10	>.99

Values are presented as means(standard deviations) or frequencies (percentage), as appropriate.
P-values comparing preterm-born children with and without PGF are calculated using the chi-square or Fisher's exact test, as appropriate.
Twenty preterm infants underwent brain MRI at term equivalent age, and abnormal MRI findings were documented in 2 children. In 1 participant from the with PGF group, subtle T2 high signal intensity in the bilateral deep white matter with focal ventricular dilatation was documented, and in another participant from the without PGF group, tiny, bilateral, T2 high signal intensity lesions in the parietal lobe deep white matter adjacent to the lateral ventricle posterior horn were noted.
BPD, bronchopulmonary dysplasia; C/S, cesarean section; GA, gestational age at birth; HCAM, histological chorioamnionitis; IVH, intraventricular hemorrhage; MRI, magnetic resonance imaging; NEC, necrotizing enterocolitis; PDA, patent ductus arteriosus; PGF, postnatal growth failure; ROP, retinopathy of prematurity; USG, ultrasonography

© 2023 Kim SY et al. *JAMA Network Open.*

eTable 3. Neurodevelopmental Outcomes in Preterm Groups, Adjusted for Gestational Age at Birth

	With PGF [N=21]	Without PGF [N=17]	*P*	$F_{1,35}$	Partial η^2	Mean difference
**FSIQ**	83.9 (17.0)	94.9 (15.8)	.005	8.76	0.200	16.63 (5.22, 28.04)
**VCI**	92.9 (15.1)	96.8 (11.0)	.13	2.41	0.064	7.35 (-2.26, 16.67)
**PRI**	82.3 (24.1)	97.8 (19.3)	.01	6.80	0.163	20.45 (4.53, 36.37)
**WMI**	87.2 (16.7)	97.0 (16.5)	.008	8.01	0.186	15.82 (4.47, 27.17)
**PSI**	79.3 (15.1)	94.1 (16.7)	.001	12.77	0.267	19.55 (8.44, 30.66)
**EF**	41.4 (8.0)[a]	44.6 (8.7)[b]	.04	4.55[c]	0.132	6.65 (0.29, 13.01)
**ATA**	63.5 (9.4)[d]	55.7 (8.0)	.001	14.08[e]	0.306	11.29 (5.16, 17.42)

Values are presented as means (standard deviations)
P-values are calculated using a general linear model with gestational age at birth as a covarying factor.
There were [a]17 children in the with PGF group and [b]16 children in the without PGF group who completed the EF test, and [d]18 children in the with PGF group who completed the ATA test.
[c]df = (1,31), [e]df = (1,33)
FSIQ, VCI, PRI, WMI, and PSI were measured using the K-WISC-IV. Executive function and attention function were measured based on the EF composite score and ATA score, respectively.
ATA, Advanced Test of Attention; EF, executive function; FSIQ, Full-Scale Intelligence Quotient; K-WISC-IV, the Korean version of the Wechsler Intelligence Scale for Children, Fourth Edition; PGF postnatal growth failure; PRI, Perceptual Reasoning Index; PSI, Processing Speed Index; VCI, Verbal Comprehension Index; WMI, Working Memory Index

© 2023 Kim SY et al. *JAMA Network Open.*

eTable 4. Diffusion Metrics and Functional Connectivity Strength for Preterm-Born Children With or Without PGF, Adjusted for Gestational Age at Birth

	With PGF [N=21]	Without PGF [N=17]	*P*	$F_{1,36}$	Partial η^2	Mean difference
**Fmajor**						
**FA**	0.498 (0.067)	0.558 (0.044)	.008	7.787	0.182	0.059 (0.016, 0.102)
**MD**[a]	9.243 (0.650)	8.709 (0.515)	.01	7.489	0.176	0.591 (0.153, 1.030)
**RATR**						
**FA**	0.462 (0.120)	0.378 (0.078)	.06	3.927	0.101	0.074 (-0.002, 0.151)
**MD**[a]	8.009 (0.652)	7.755 (0.326)	.02	6.395	0.154	0.310 (0.061, 0.559)
**LILF**						
**FA**	0.498 (0.053)	0.542 (0.034)	<.001	15.775	0.311	0.060 (0.030, 0.091)
**MD**[a]	8.658 (0.387)	8.278 (0.371)	.003	10.449	0.230	0.442 (0.164, 0.720)
**LSLFP**						
**FA**	0.389 (0.050)	0.431 (0.045)	.02	5.645	0.139	0.041 (0.006, 0.077)
**MD**[a]	8.312 (0.318)	7.902 (0.455)	.002	11.279	0.244	0.467 (0.185, 0.749)
**LSLFT**						
**FA**	0.382 (0.049)	0.421 (0.043)	.04	4.736	0.119	0.037 (0.002, 0.071)
**MD**[a]	8.402 (0.294)	8.070 (0.352)	.001	13.235	0.274	0.411 (0.182, 0.641)
**Seed or target**						
**Precuneus**						
**Left SFG**	0.035 (0.156)	0.186 (0.165)	.04	4.497	0.114	0.122 (0.005, 0.238)
**Left MidFG**	0.074 (0.211)	0.193 (0.148)	.06	3.862	0.099	0.133 (-0.004, 0.270)
**AC**	0.134 (0.202)	0.086 (0.111)	.27	1.265	0.035	-0.068 (-0.192, 0.055)
**PC**	0.605 (0.309)	0.639 (0.302)	.97	0.001	0.000	0.004 (-0.222, 0.229)
**PCC**						
**AC**	0.055 (0.162)	0.142 (0.191)	.26	1.299	0.036	0.073 (-0.057, 0.202)
**Right SPL**	-0.075 (0.155)	-0.090 (0.188)	.84	0.042	0.001	0.013 (-0.112, 0.137)
**Left SPL**	-0.081 (0.144)	-0.112 (0.148)	.80	0.067	0.002	0.013 (-0.089, 0.115)
**Right sLOC**						
**Right SPL**	0.156 (0.273)	0.236 (0.278)	.20	1.776	0.048	0.131 (-0.069, 0.331)
**Left SPL**	0.152 (0.267)	0.286 (0.209)	.05	4.338	0.110	0.181 (0.005, 0.357)
**Left sLOC**						
**Right SPL**	0.127 (0.286)	0.231 (0.276)	.06	3.956	0.102	0.192 (-0.004, 0.389)
**Left SPL**	0.271 (0.316)	0.385 (0.277)	.07	3.579	0.093	0.197 (-0.014, 0.409)

Values are presented as means (standard deviations).
P-values are calculated using a general linear model with post hoc Bonferroni test.
AC, anterior division of the cingulate gyrus; Fmajor, forceps major of corpus callosum; LILF, left inferior longitudinal fasciculus; LSLFP, left superior longitudinal fasciculus-parietal bundle; LSLFT, left superior longitudinal fasciculus-temporal bundle; MidFG, middle frontal gyrus; PC, cingulate gyrus, posterior division; PCC, posterior cingulate cortex, PGF, postnatal growth failure; RATR, right anterior thalamic radiation; SFG, superior frontal gyrus; anterior division; sLOC, superior lateral occipital cortex; SPL, superior parietal lobule
[a]Originallly calculated as millimeter squared per second and rescaled 100 times as MD x 10000

© 2023 Kim SY et al. *JAMA Network Open.*
