# Supplementary material for: Association of Brain Microstructure and Functional Connectivity With Cognitive Outcomes and Postnatal Growth Among Early School–Aged Children Born With Extremely Low Birth Weight
Source: JAMA Netw Open. 2023 Mar 2;6(3):e230198. doi: 10.1001/jamanetworkopen.2023.0198 (PMC9982697; doi:10.1001/jamanetworkopen.2023.0198)
Supplement: Supplement 2. — Data Sharing Statement [file jamanetwopen-e230198-s002.pdf]

Data Sharing Statement

Kim. Association of Brain Microstructure and Functional Connectivity With Cognitive Outcomes and Postnatal Growth Among Early School-Aged Children Born With Extremely Low Birth Weight. *JAMA Netw Open*. Published March 02, 2023.
doi:10.1001/jamanetworkopen.2023.0198

Data
Data available: No
